# Research on Emotion Recognition Method Based on Adaptive Window and Fine-Grained Features in MOOC Learning

**DOI:** 10.3390/s22197321

**Published:** 2022-09-27

**Authors:** Xianhao Shen, Jindi Bao, Xiaomei Tao, Ze Li

**Affiliations:** 1 School of Information Science and Engineering, Guilin University of Technology, Guilin 541004, China; 2 Guangxi Key Lab of Multi-Source Information Mining and Security, Guangxi Normal University, Guilin 541004, China

**Keywords:** emotion recognition, eye movement signal, audio and visual features, adaptive window, fine-grained feature

## Abstract

In MOOC learning, learners’ emotions have an important impact on the learning effect. In order to solve the problem that learners’ emotions are not obvious in the learning process, we propose a method to identify learner emotion by combining eye movement features and scene features. This method uses an adaptive window to partition samples and enhances sample features through fine-grained feature extraction. Using an adaptive window to partition samples can make the eye movement information in the sample more abundant, and fine-grained feature extraction from an adaptive window can increase discrimination between samples. After adopting the method proposed in this paper, the four-category emotion recognition accuracy of the single modality of eye movement reached 65.1% in MOOC learning scenarios. Both the adaptive window partition method and the fine-grained feature extraction method based on eye movement signals proposed in this paper can be applied to other modalities.

## 1. Introduction

The massive open online course (MOOC) has become a popular learning model. MOOCs have grown rapidly since 2012 [1]. Compared to traditional classes, the core advantage of MOOCs is that is they are not limited by space, and they can reduce the gap in teaching resources between different regions and improve teaching levels. According to China Education Daily, MOOCs have provided important support for 250 million students during the pandemic.

However, in MOOCs, students and teachers cannot communicate face to face, and, as a result, teachers are unable to provide timely feedback on students’ different emotions during the learning process [2]. As a result, there is an urgent need to resolve the problem of identifying students’ emotions in MOOC learning. Some studies have shown that the challenge of recognizing students’ emotions is that the emotional fluctuation in the learning process is low. Julia Moeller [3] found that most students have negative emotions in the process of learning. Shan Li [4] found that the negative emotions in the learning process had less emotional fluctuation. Tiina Törmänen [5] found that, due to the lack of teacher–student interaction and collaborative learning between classmates in online learning, students’ learning enthusiasm and emotional fluctuation were low. Weak emotional fluctuation leads to inconspicuous characteristics for students in different emotional states. To solve this problem, we propose a method to identify learner emotion by combining eye movement features and scene features. This method uses an adaptive window to partition samples and enhances them through fine-grained feature extraction. The method is divided into two steps. First, an adaptive window is used to segment the sample according to the fluctuation cycle of emotion, and then fine-grained feature extraction is performed on the sample to extract the temporal relationship. The adaptive window in the first step makes the emotion fluctuation in the sample more obvious and improves the sample quality. The fine-grained feature extraction in the second step can increase the discrimination between samples. MOOCs require students to watch videos for a long time, so eye movement signals can be used as an important basis for student emotion recognition. In addition, unlike other physiological signals, the acquisition of eye movement signals does not require students to wear sensors, which means they will be unaffected by such sensors and not distracted. Scenario features include audio features and video image features in MOOC videos. The audio and video images of MOOC video, which are sources of stimulation of students’ emotions, are an important basis for identifying students’ emotions. If the audio is dull or the video images are monotonous, students may feel bored; if the audio is interesting or the video image content is rich, it is easy to generate positive emotions in students [6].

The important contributions of this paper are as follows: (1) a sample partition method based on an adaptive window is proposed. The method extracts the fluctuation cycle of emotion states from the changes in eye movement signals as the window of a sample partition, which can make the emotion fluctuation in the sample more complete. Compared with the conventional static window and the adaptive window applied in other tasks, the method proposed in this paper has better performance and is more suitable for application in MOOC video learning scenarios. (2) The second contribution addresses the problem that the features of learners in different emotion states are not obvious, which leads to the high similarity of samples. In this paper, a fine-grained feature extraction method is proposed. In this method, each sample is finely divided into several sub-samples, the features of each sub-sample are extracted, and sub-sample features of the same type can form a feature curve. Compared with the original features, the feature curve contains more information and changes, which can increase the distinction between samples. (3) TCN is proposed to construct the model and, compared with the conventional timing model LSTM, the results show that the TCN model is more suitable for MOOC scenarios.

## 2. Related Work

### 2.1. Research on Emotion Recognition in MOOC Scenarios

At present, emotion recognition tasks in MOOC scenarios can be divided into emotion recognition based on after-class comments, emotion recognition based on learners’ physiological signals and emotion recognition based on facial expressions. Ye et al. [7] proposed a MOOC learning emotion recognition method based on transfer learning. This method used two hierarchical attention networks to complete the transfer of emotional features, and the recognition precision of learners’ positive and negative emotions reached 85.9%. Atapattu et al. [8] proposed machine learning models based solely on language and discourse features extracted from learners’ discussion posts. In the confused state recognition task for learners, the F1 score reached 83%. Nandi et al. [9] proposed a real-time emotion classification system (RECS)-based logistic regression (LR) model trained in an online fashion using the stochastic gradient descent (SGD) algorithm. This method can classify emotions in real time by training the model online using EEG signals, and the average recognition accuracy of eight emotions reached 47.75%. Jason et al. [10] improved the facial expression recognition network FaceLiveNet and added Dense, and the average recognition accuracy of six emotions reached 69.99%. There is a serious delay in the text recognition of after-class comments, and the status of students cannot be obtained in a timely fashion. In the task of emotion recognition using EEG signals, wearing sensors on the head distract students and makes the obtained data noisy. In an emotion recognition task based on facial expressions, students’ facial expressions do not change significantly during the learning process, which makes the classification effect not ideal. Therefore, the emotion recognition method based on eye movement, audio and video images proposed in this paper can effectively solve the problems existing in the research on emotion recognition in MOOC learning scenarios.

### 2.2. Research Based on Adaptive Window

At present, fixed-length static windows are used to partition the samples in emotion classification tasks. Zhang et al. [11] used a 10 s static window to partition an EEG signal and eye movement signal; Han et al. [12] partitioned audio and visual features into 8 s static samples; Zhou et al. [13] partitioned video and audio streams into 40 ms static samples. In past research, several medical and assisted-driving studies, as well as other kinds of studies, have used adaptive windows. Yamamoto et al. [14] proposed a heart rate estimation method based on multi-classification signals, which used an adaptive window to reduce errors in peak detection. Experimental results showed that this method was superior to existing signal classification methods. Sun et al. [15] proposed an improved sliding window method to detect the peaks and troughs of PPG signals, which improved the quality of PPG signal samples. Yang et al. [16] proposed a feature extraction method based on adaptive sliding windows to capture semantic features in sentences through adaptive windows, and this method could accurately distinguish deception information from rule information. Li et al. [17] proposed an adaptive correlation window energy detection algorithm, which significantly reduced the signal-to-noise ratio. Gao et al. [18] proposed a driving assistance model, DMPM, which could dynamically identify the optimal sliding window size of input data and had significantly better performance than other models. The current research results show that the accuracy of recognition can be effectively improved by using adaptive windows in different tasks. However, few studies have applied adaptive windows to emotion recognition tasks. Therefore, this paper proposes a method of partitioning samples by using the cycle of emotion fluctuation as an adaptive window.

The subsequent sections are as follows. The third section introduces the dataset collection, the data preprocessing and the method used in this paper. In Section 4, experiments carried out to verify the proposed method are described. In Section 5, the experiments to verify the effectiveness of our method with the public HCI-Tagging Databases and a comparison with other methods are described. Section 6 is the conclusion.

## 3. Materials and Methods

### 3.1. Dataset Collection

In the data collection stage, four learning videos related to ancient Chinese history, physics, Tang poetry and the pyramids were gathered. Each video lasted about two and a half minutes. Eye movement signals were collected with the eye movement instrument Tobii-TX300. The sampling frequency was 60 Hz/s, and the 59 subjects were college students aged between 20 and 23 years. The male to female ratio was close to 1:1, and all of them had signed informed consent. The subjects watched the videos after pupil calibration in a laboratory environment with constant brightness. When the subjects felt interested, happy, confused or bored during watching the video, they pressed the corresponding keys on the keyboard to mark the emotion they were feeling. After learning, the subjects reviewed the study videos and videos of their facial expressions during the study and expand the marked point into an emotional event. The emotional intensity was rated on a scale from 5–1 from strong to weak. The original experimental dataset was obtained by extracting synchronous eye movement signal, audio and video image data. Eye movement signals included pupil diameter, gaze points (the pixel coordinates of the eye on the screen were calculated with Pupil-CR technology [19]) and eye states, such as blinking, fixation and saccade. Audio signals were extracted using the Mel Frequency Cepstrum Coefficient (MFCC), and video images were image frames extracted from the aforementioned videos. In Section 4.2, the features extracted from eye movement signals, audio signals and video images are described.

### 3.2. Feature Extraction from Eye Movement, Audio and Video Images

To solve the problem of eye movement signal data loss, we used a linear fitting method to complete the pupil diameter data. Eye movement signals are physiological signals, and everyone has different pupil diameters in the calm state. Therefore, we calculated the pupil diameters for all subjects in the calm state as the baseline value. The pupil diameter in each frame in the dataset was subtracted from the baseline value to eliminate differences in pupil diameter between subjects in the calm state.

In the feature extraction process, the pupil diameter was the main data, and the pupil diameter and its first-order-difference time-domain characteristics and waveform characteristics, as well as saccade times and fixation time, were extracted from the samples. The adopted eye movement features are listed in Table 1.

In the scenario feature extraction, there were two modalities: audio and video image. In the audio modality, the MFCC coefficient of the audio signal was obtained by filtering, and the time domain features of the MFCC coefficient and its first-order difference were extracted. In the video image modality, the pixel change rate Z of the two adjacent frames was calculated, as shown in Formula (1):

(1) $$Z=\frac{\sum_{\left(i,j\right)\in A_{t}}A_{t}\left(i,j\right)}{n}$$

In Formula (1), $A_{t}=X_{t}-X_{t-1}$ is the matrix of difference between two adjacent grayscale images. *n* is the total number of pixels in the gray image. We calculated the value of *Z* for every two adjacent images in the window to get a sequence of pixel change rate *Zseq =*$\;\left[Z_{1},Z_{2},\cdot \cdot \cdot \cdot \cdot \cdot ,Z_{u-1}\right]$, where u is the number of image frames in the window. The time domain features of sequence *Z* were obtained. The audio and video features utilized are shown in Table 1. The features extracted from SWS and AWS in the subsequent experiments are all listed in Table 1.

### 3.3. Process of Methods

**Figure 1:**
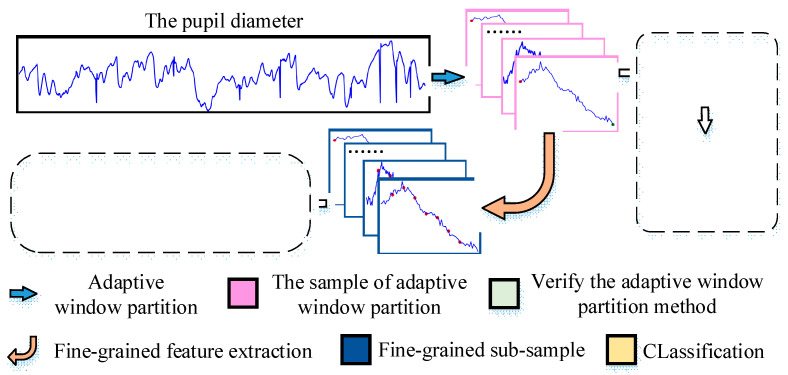
The overall process of our method.

The overall process of our method is shown in Figure 1. The pupil diameter data were partitioned into samples using our adaptive window partition method. The effectiveness of our adaptive window partition method was verified. Fine-grained feature extraction was carried out on the partitioned samples, and the fine-grained sub-samples were obtained. The features in the sub-samples were extracted and classified by a time-series model.

#### 3.3.1. Sample Segmentation Based on Adaptive Window

Studies have shown a relationship between pupil diameter and different emotional states. Ai Li found that pupil diameter decreased when people were suffering and increased during negative and positive emotional states [20]. Laxmipriya Moharana found that the pupil diameter increased in states of surprise, sadness and happiness [21]. Pupil diameter changes with the ups and downs of emotion. In the process of emotional development from production to enhancement to attenuation, the pupil diameter also produces a cycle of fluctuation [22].

**Figure 2:**
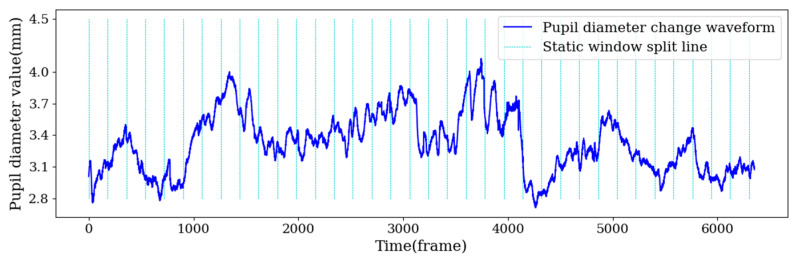
Schematic diagram of pupil diameter partitioning samples according to 3 s static window.

The whole cycle of fluctuation moves from peak to peak or from trough to trough. Students have many emotional fluctuations in the process of watching videos, but using a static window to partition samples will destroy the complete fluctuation cycle. Figure 2 shows the fluctuation of pupil diameter in the process of watching the learning video, in which the horizontal axis is time, the vertical axis is the pupil diameter, the sampling frequency is 60 Hz/s and the green dotted line represents the 3 s static window, and each window was partitioned to obtain a sample. As shown in Figure 2, some of the data samples segmented by the 3 s static window contained a complete cycle and some adjacent cycle bands, some contained only one cycle part of the band and a few samples were complete cycles. Such samples cannot effectively reflect the changes in learners’ emotions. Therefore, a sample partition method based on an adaptive window is proposed in this paper. In the following sections, the adaptive window partition sample is abbreviated AWS, and static window partition sample is abbreviated SWS.

**Figure 3:**
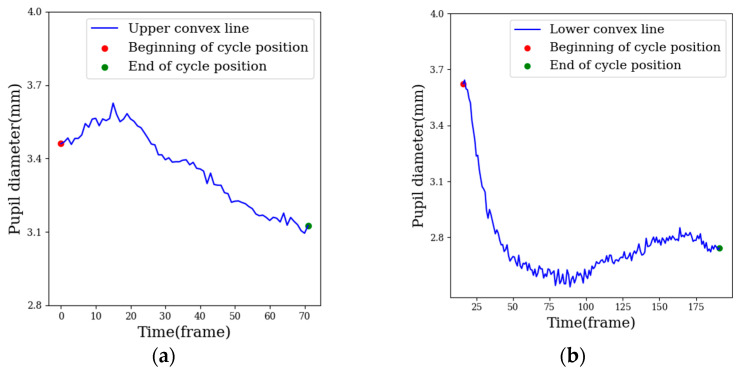
Cycle curve, (**a**) upper convex line, (**b**) lower convex line.

The sample partition method based on an adaptive window partitioned a complete fluctuation cycle as a window. The range of a window was from the beginning position of the fluctuation cycle to the end position. In emotion fluctuations, emotional arousal usually increases at first and then decreases, so it is assumed that the pupil diameter increases with the increase in emotional arousal and decreases with the decrease in emotional arousal, indicating that the cycle curve is an upper convex line, as shown in Figure 3a. Alternatively, the pupil diameter decreases with the increase in emotion, indicating that the cycle curve is a lower convex line, as shown in Figure 3b. The start point and end point of the upper convex line are the local minimums across the whole band, and the start point and end point of the lower convex line are the local maximums across the whole band.

**Figure 4:**
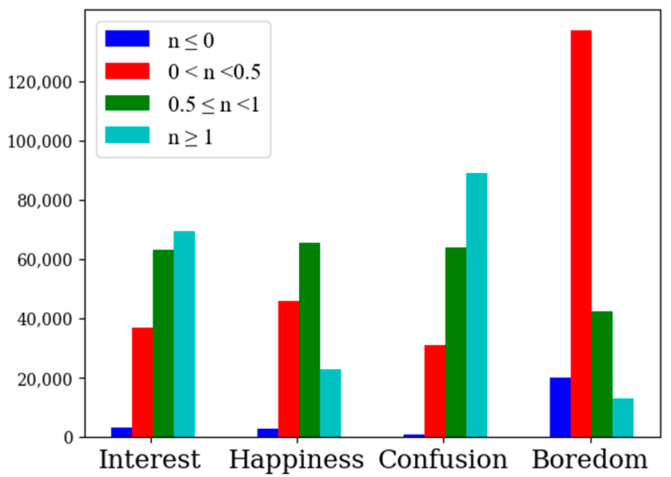
Distribution map of pupil diameter difference in four emotional states.

In order to determine whether to use the upper convex line or the lower convex line for adaptive window division, we calculated the difference between the pupil diameter in each frame and the baseline value of the pupil diameter in the quiet state, as shown in Figure 4, where blue, red, green and cyan represent the numbers of difference frames in four intervals ((−∞, 0], (0, 0.5), [0.5, 1) and [1, +∞)). As can be seen from the graph, the pupil diameter difference in the interested state was mainly distributed in (0.5, +∞), the pupil diameter difference in the happy state was mainly distributed in (0, 1), the pupil diameter difference in the confused state was mainly distributed in (0.5, +∞) and the pupil diameter difference in the bored state was mainly distributed in (0, 0.5). Values for the pupil diameter difference that were greater than zero in the four emotional states of interest, happiness, confusion and boredom, were 98.2%, 98%, 99.5% and 90.7%, respectively. The pupil diameters in the four emotional states were larger than in the calm state, indicating that the pupil diameter increased with the enhancement of the four emotional states. Therefore, the upper convex line was used for adaptive window division, and the beginning and end positions of the cycle were the local minimum values across the whole process of pupil diameter change.

Although the proportion of pupil diameter difference values greater than zero in the four emotional states was more than 90%, the proportion of pupil diameter difference values in the bored state was significantly lower than that of the other three emotional states. The distribution for the proportion of pupil diameter difference values less than zero in the three emotions of interest, happiness, and confusion was within 2%, while the distribution for the proportion of pupil diameter difference values less than zero in the state of boredom was 9.3%, and the pupil diameter difference distribution in the state of boredom was mostly in the range of 0 to 0.5 (extremely close to zero). Since errors existed for all four emotions, we excluded the influence of error on the distribution of the differences in pupil diameter in the bored state. It was judged that the change in pupil diameter in the bored state did not fluctuate much and was obviously different from the other three emotions. When using an adaptive window to partition samples, it is necessary to obtain the position of the partition point, which is the end point of the current window and the beginning point of the next window. The process of calculating the location of the partition point is shown in Algorithm 1, where n is the minimum length of adaptive sample, and the length of adaptive window can be controlled by controlling the value of n. We set the first frame collected as the start point of the first window and started traversing after the nth frame of the start point. If the pupil diameter value of the current frame was the smallest in the first n frames and the last n frames, the current position was considered as a local minimum across the whole band and as the end point of the current window and the beginning point of the next window.
**Algorithm 1** Calculating the partition points of the adaptive window**Input:** List of pupil diameter during viewing video: *Pd*
**Output:** Split point list: *Sp***Initialize:** *Sp*=None  for i = n to the length of *Pd* do   i is the index of *Pd*   if *Pd*[i] < the Minimum of *Pd*[i-n:i] and *Pd*[i] < the Minimum of *Pd*[i+1:i+n] then    i is the local minimum from i-n to i+n    Deposit i into *Sp*    i =i+n   else    i=i+1   end ifend for

**Figure 5:**
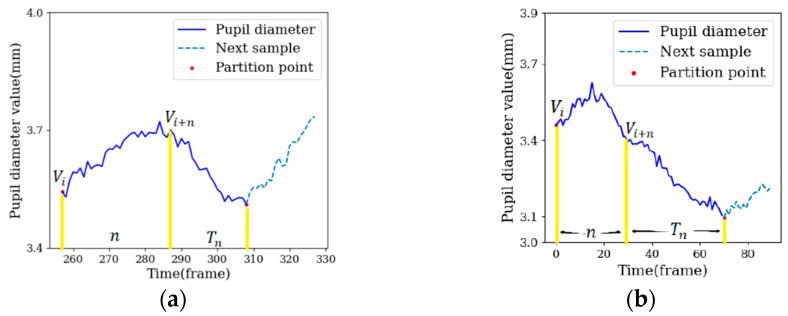
Schematic diagram of adaptive window length, where (**a**) shows $T_{n}$ less than *n* and (**b**) shows $T_{n}$ greater than *n*.

*n* was only the theoretical minimum of the length of adaptive windows, and most of the lengths of the adaptive windows were distributed around 2*n*. Taking an adaptive window as an example, the constraint conditions of the starting point position *i* are shown in Formula (2), where $V_{i}$ represents the pupil diameter value of starting point *i*, $\mathrm{Min}\left(V_{i-n},V_{i-1}\right)$ represents the minimum value within the interval [$V_{i-n},V_{i-1}$], and $\mathrm{Min}\left(V_{i+1},V_{i+n}\right)$ represents the minimum value within the interval [$V_{i+1},V_{i+n}$]. If the length of the adaptive window is *n*, the end point is at the position *i + n*, indicating that $V_{i+n+1}$ is the minimum value within the interval [$V_{i+1},V_{i+n}$]. However, $V_{i}$ is also the minimum value within the interval [$V_{i+1},V_{i+n}$], which does not conform to the rule of change for the pupil diameter. The cycle curve for the pupil diameter was an upper convex line, and the rule of change from the start point to the end point was that the pupil diameter rises first and then falls. It was difficult for the pupil diameter to fall to the local minimum in *n* frames. Therefore, the length of the adaptive window was calculated with Formula (3), where $A_{len}$ represents the true length of the adaptive window, $T_{n}$ represents the length of the pupil diameter from position *i + n* to the end point and $T_{n}$ was obtained by recording the number of traversals in Algorithm 1. Figure 5 shows the adaptive window under the condition of *n* = 30. When the pupil diameter increased for a short time, the local minimum could be obtained within 2*n* frames and $T_{n}$ was smaller than *n*, as shown in Figure 5a. When the pupil diameter increased for a long time, the local minimum could not be obtained within 2*n* frames and traversals continued until it dropped to the local minimum. In this case, $T_{n}$ was bigger than *n*, as shown in Figure 5b. Thus, the lengths of most adaptive windows were distributed around 2*n*.

(2) $$\left\{\begin{array}{c} V_{i}<Min\left(V_{i-n},V_{i}\right)\\ V_{i}<Min\left(V_{i+1},V_{i+n}\right) \end{array}\right.$$

(3) $$A_{len}=n+T_{n}$$

**Figure 6:**
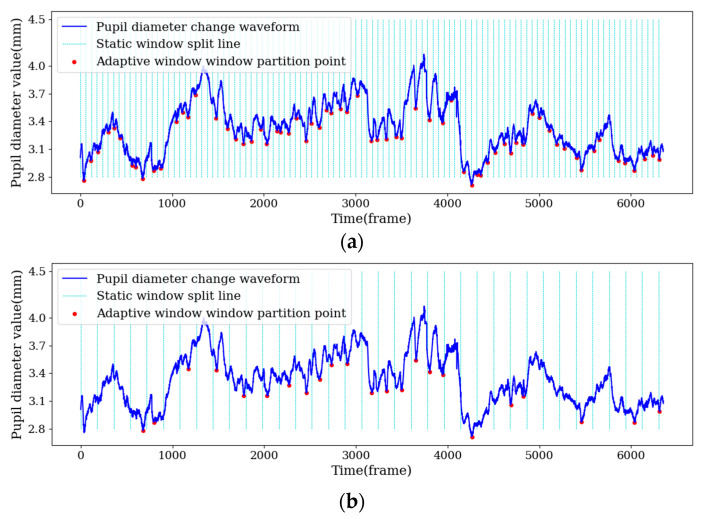

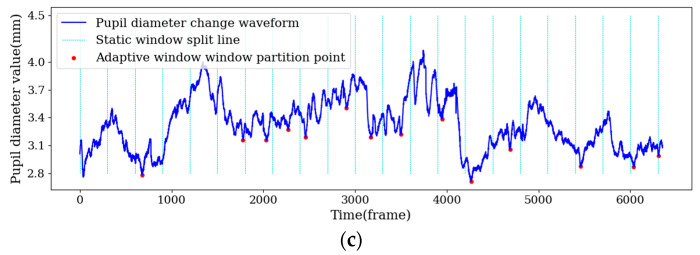
AWS and SWS pupil diameter diagrams: (**a**) 1 s static window and adaptive window for n = 30, (**b**) 3 s static window and adaptive window for n = 90, (**c**) 5 s static window and adaptive window for n = 150.

Therefore, the AWSs with n = 30/90/150 were respectively extracted to correspond to SWSs with static window lengths of 1 s/3 s/5 s (60 frames/180 frames/300 frames), as shown in Figure 6.

#### 3.3.2. Fine-Grained Feature Extraction

**Figure 7:**
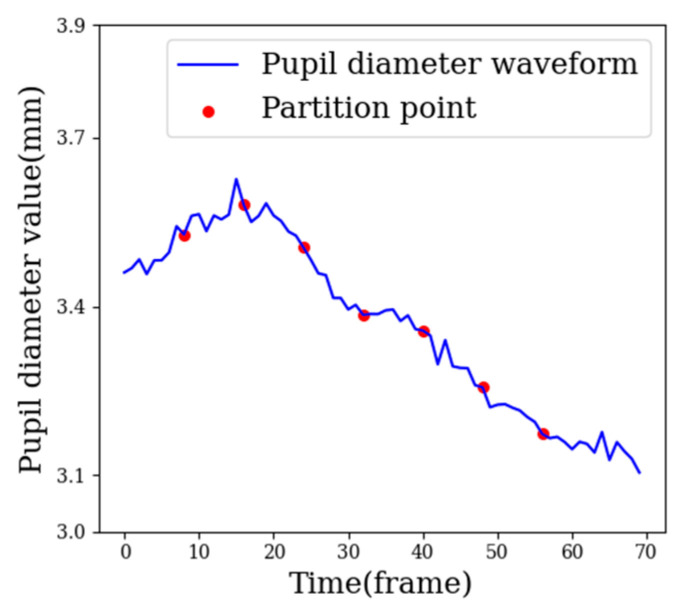
Fine-grained partition of adaptive samples.

As the emotional state of learners in the MOOC video learning scenarios did not change significantly, the similarity between samples was too high. In order to enhance the differences between samples, finer-grained feature extraction was carried out on samples. A sample was divided into eight fine-grained sub-samples on average, as shown in Figure 7. The eight fine-grained sub-samples formed a sub-sample sequence according to the segmentation order. The same features of the original sample were extracted from the eight fine-grained sub-samples. The same features in the fine-grained sub-samples could form a feature curve in order, and each feature value in the original sample could be replaced by a corresponding feature curve. The specific process is as follows.

As can be seen from Figure 7, the samples were evenly divided into eight parts, and each part was treated as a small, fine-grained sub-sample, as shown in Formula (4):

(4) $$A=\left(FG_{1}\to FG_{2}\to \cdots \cdots \to FG_{8}\right)$$

where *A* is the original sample, *FG* is the fine-grained sub-sample and → indicates that the two samples are connected according to the temporal relationship.

These eight fine-grained subsamples constituted a full sample based on the temporal relationship. In each fine-grained sub-sample, the same features as the original sample were extracted. The same features in the eight fine-grained sub-samples could form a feature curve to replace the corresponding feature in the original sample, as shown in Formula (5):

(5) $$\left\{\begin{array}{c} FGF_{m}=\left[f_{1},f_{2}\cdots \cdots ,f_{n}\right]\\ FC_{t}=\left[FGF_{1}\left[t\right],\cdots \cdots ,FGF_{8}\left[t\right]\right] \end{array}\right.$$

where *FGF* represents the feature set extracted from the fine-grained sub-sample, *f_n_* represents a single feature in the fine-grained sub-sample, m represents the index of the fine-grained sub-sample (the value of *m* is 1–8) and n represents the total number of features extracted from the sample. *FC* represents the feature curve and *t* represents the index of the feature in the fine-grained sub-sample and the index of the corresponding feature curve (the value of *t* is 1–*n*).

Therefore, conventional feature extraction methods can extract n features from samples, while fine-grained feature extraction of samples can extract n feature curves, as shown in Formula (6), where F represents the feature set in the sample, FC represents the feature curve extracted from the fine-grained sample and $f_{n}$ represents the feature value extracted from AWS:

(6) $$F=\left\{\begin{array}{c} \left[FC_{1},\cdots \cdots ,FC_{n}\right],\;\mathrm{Fine grained sample}\\ \left[f_{1},f_{2}\cdots \cdots ,f_{n}\right],\;\;\;\;\;\;\;\;\;\;\;\;\;\;\;\;\;\;\;\;\;\mathrm{AWS}\; \end{array}\right.$$

**Figure 8:**
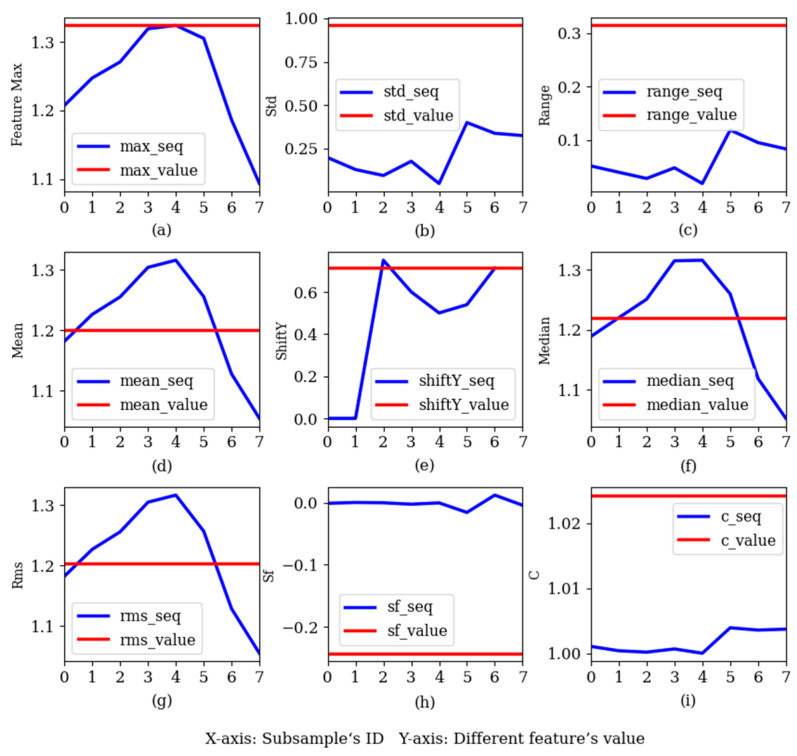
A comparison of the feature values in the AWS with the feature curves in the fine-grained samples. (**a**) Maximum, (**b**) standard deviation, (**c**) range, (**d**) average, (**e**) average fluctuation in pupil fixation in the vertical direction, (**f**) median, (**g**) root mean square, (**h**) skewness factor, (**i**) kurtosis factor.

Several features in the eye movement signal were selected for visual analysis, as shown in Figure 8, where the red line represents the value of the features in the AWS, and the blue broken line is the feature curve, which is composed of features from eight fine-grained subsamples. The x-axis is the ID of the subsample sequence, and the y-axis is the feature value.

It can be seen from the figure that the feature curves could contain more changes than the discrete feature values. The fine-grained feature method could extract more feature information and increase the distinction between samples, and the feature curve contained time series features, which were well-suited for the input of the time series model. Conventional methods of extracting features cannot keep time series information in the original data. However, the fine-grained feature extraction method can extract features and time series information between features.

## 4. Experiment

### 4.1. Comparison of AWS with SWS

The correlation between sample features and sample labels is an important measure of sample quality. Taking a 1 s SWS and AWS (*n* = 30) as examples, the correlation between all features and emotion labels can be calculated. Figure 7 shows the correlation between the features extracted from the AWS and sentiment labels, and the correlation between the features extracted from the SWS and sentiment labels. Features 0 to 18 are time-domain features of the pupil diameter, features 19 to 32 are time-domain features of the first-order differences in the pupil diameter, features 33 to 54 are waveform features of the pupil diameter and features 55 to 70 are waveform features of the first-order differences in the pupil diameter. Features 71 to 91 are the time-domain features of the audio signal, 92 to 112 are the time-domain features of the first-order differences in the audio signal and 113 to 123 are the time-domain features extracted from the video image. As can be seen from the figure, in the AWS (*n* = 30), the correlations between most features and emotion labels was higher than those between features and emotion labels in the 1 s SWS.

**Figure 9:**
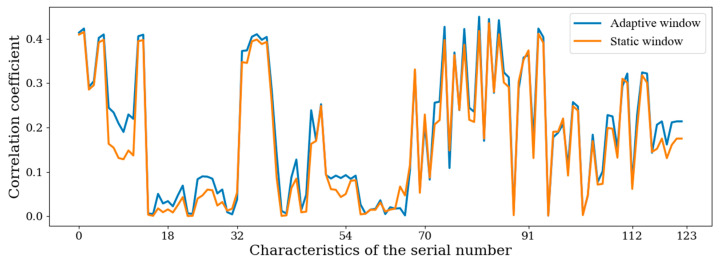
Correlation coefficients between features and emotional labels.

The average values for the correlation coefficients between different types of features and emotion labels in the AWS and SWS are shown in Table 2. The table shows that the correlations between the various feature types and emotion labels in the AWS were higher than in the SWS. It can be seen from Table 2 and Figure 9 that using the adaptive window to segment the sample could make the emotion fluctuation in the sample more obvious, so the quality of the AWS was higher than that of the SWS.

The sample recognition effect directly measures the quality of the samples. The data for 10 subjects were selected from among the 59 subjects as the test set, and the remaining 49 subjects were used as the training set. The features extracted from the training set and the test set were normalized, and then the normalized data were reduced by PCA to retain the principal components, the contribution rate of which was greater than 1%. The eye movement signal was reduced to 16 principal components, the audio signal was reduced to 8 principal components, and the video image was reduced to 3 principal components.

**Figure 10:**
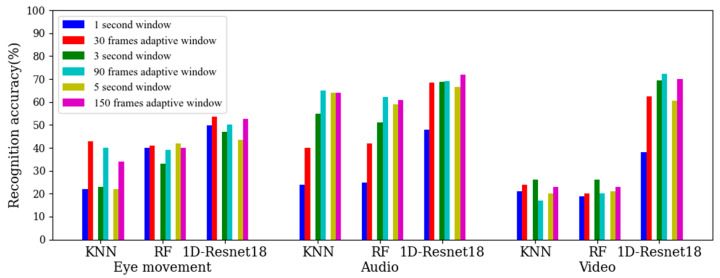
The recognition accuracies for the AWSs and SWSs.

KNN, random forest and 1D-Resnet18 were selected as classifiers. The 1 s static window corresponded to an adaptive window with n = 30, the 3 s static window corresponded to an adaptive window with n = 90 and the 5 s static window corresponded to an adaptive window with n = 150. The recognition accuracies for the AWSs and SWSs are shown in Figure 10.

According to Figure 10, compared to the static window partition method, the adaptive window partition method proposed in this paper can improve the recognition accuracy for different sizes of windows, different classification models and different modalities. In the 1 s SWS, the recognition accuracy for the eye movement signal was high, but the effects for the audio signal and video image were poor; as the window was too small and the length was fixed, it was difficult to extract effective information from it. In the AWS with n = 30, the variable length window made the audio features and video image features more obvious, so the recognition effect was greatly improved. According to Table 2 and Figure 10, the quality of the AWS was better than that of the SWS.

Table 3 shows the results of the comparison between the adaptive window partition method proposed in this paper and the adaptive window partition method proposed in [14] and [15]. The evaluations in the Table 3 are for accuracy (Acc), macro-F1 (m-F1) and area under ROC curve (AUC). The classification models all used 1D-Resnet18. As can be seen from Table 3, compared with the adaptive window partitioning methods proposed in other papers, our method is more suitable for emotion recognition tasks. The results show that taking the period of emotional fluctuation as the window to partition the sample can make the emotional changes in the sample more obvious.

### 4.2. Comparison of Fine-Grained Feature Methods

Feature curves extracted from fine-grained samples contain temporal relations. In order to verify the effects of the feature curves, the temporal convolutional network (TCN) [23] and LSTM were selected as classification models. LSTM is a popular time-series model, TCN is a newer and less widely used time-series model, but TCN can mine the deep information from the features. In this task, eight fine-grained sub-samples represented eight moments from which features were extracted, such as Max1–Max8 (feature curve in Figure 8a). If n features are extracted from a sub-sample, the number of input channels is n, and the input format is [batch_size, n, 8].

The features extracted from the fine-grained sub-samples were normalized, and then the normalized data were reduced by PCA to retain the principal components with contribution rates greater than 1%. The eye movement signal was reduced to 10 principal components, the audio signal was reduced to 11 principal components and the video image was reduced to 3 principal components.

As combinations of temporal networks and convolutional networks are adopted in many studies, four models (TCN, TCN + CNN, LSTM and LSTM + CNN) were used to verify the accuracy of the three modalities. In addition, feature layer fusion was used to verify the effect of combining eye movement features with scenario features, and the results are shown in Table 4. Since the features in the AWS did not contain a temporal relationship, the classifier in the AWS only used a machine learning model and convolution model.

It can be seen from Table 4 that the recognition accuracy of the AWS was significantly improved after fine-grained feature extraction. The three modalities of eye movement, audio and video image were significantly improved, which preliminarily proved the effectiveness of fine-grained feature extraction. This was further verified through analysis of the confusion matrix and the ROC curve and comparison with public datasets. The recognition accuracy of the TCN model was significantly better than that of LSTM. The recognition accuracy of the TCN + CNN model was significantly better than that of LSTM + CNN. This was because TCN is better for capturing sequential dependencies, and it uses convolution to capture local information, so the recognition effect of TCN is better than LSTM. Using feature layer fusion, the method involving eye movement features and scenario features fusion can improve the accuracy of learners’ emotion recognition.

**Figure 11:**
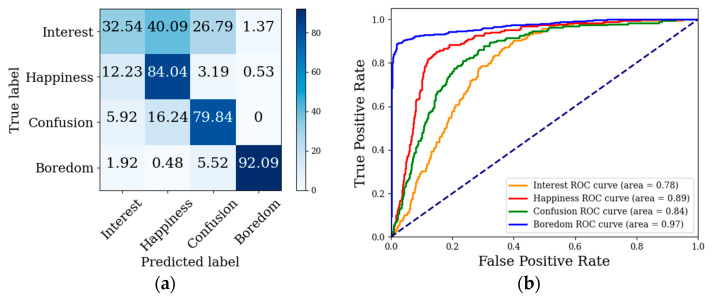
Confusion matrix and ROC curve for feature-level fusion model: (**a**) confusion matrix, (**b**) ROC curve of decision-level fusion.

The confusion matrix and ROC curve of the model after feature level fusion are shown in Figure 11. As shown in Figure 11a, the model had the best recognition effect for boredom and the worst recognition effect for interest, as was also indicated by the ROC curve in Figure 11b. Interest was easily identified as happiness and confusion. In terms of eye movement signals, students in interested, happy and confused states were all watching the learning video carefully, and the pupil diameter showed similar behavior. In the bored state, students’ attention diverged, and the pupil diameter was significantly different from the other three states. In terms of scene features, the videos that made students interested usually made students happy and confused, but the videos that made students confused did not necessarily make students interested. Thus, interest is easier identified as happiness than confusion.

## 5. Discussion

The public dataset HCI was used to further validate our method. The public HCI-Tagging Databases [24] were developed with 30 volunteers from different cultures and educational backgrounds, including 17 women and 13 men. Nine emotions—neutral, anger, disgust, fear, happiness, sadness, surprise, delight and anxiety—were induced by watching 20 movie clips. Six subjects were selected and their experimental data were used as the test set, and the remaining 24 subjects were used as the training set. The data from the training and test sets were normalized and then reduced in dimensions.

Taking the AWS with *n* = 90 as an example, the fine-grained features were extracted. LSTM, TCN, LSTM + CNN and TCN + CNN were then used to verify the fine-grained feature extraction method, as shown in Table 5.

As can be seen from Table 5, after fine-grained feature extraction with the AWS, the recognition accuracy for the three modalities was improved, and the eye movement modality showed the best improvement effect. The recognition accuracy for the eye movement modalities was improved by 12.7%. The recognition accuracy for the video image modalities was improved by 4.6%. The recognition accuracy for the audio modalities was improved by 0.2%. In general, the results for the public dataset were consistent with those for our own dataset, which further validates our approach. After extracting the fine-grained features of the samples, the distinction between samples could be enhanced, making them more conducive to classification. From the perspective of the model, the recognition accuracy of TCN was better than that of LSTM, and the recognition accuracy of TCN + CNN was better than that of LSTM + CNN, which indicates that TCN is more suitable for emotion recognition tasks.

Because the modalities were different, we only used the eye movement modality for the comparison with the methods in other papers. The evaluations in Table 6 are for accuracy (Acc), macro-F1 (m-F1), area under ROC curve (AUC), floating-point operations (FLOPs), and the number of parameters (NPs). The FLOPs are the numbers of floating-point operations in one training turn, which were used to measure the time complexity of the model. One MFLOP equals one million FLOPs. NPs represent the total numbers of parameters inside the model, which were used to measure the size of the model. The classification model used in [25] was SVM without NPs.

Table 6 shows a comparison with other methods. Our method achieved the best results for the three measures of evaluating model identification performance (Acc, F1 score and AUC). However, in the two evaluation indexes for complexity analysis, FLOPs only outperformed SVM, while the NPs were the highest among all methods. As the TCN model has many parameters, the complexity of the model is high.

## 6. Conclusions

In this paper, a new adaptive window partition method is proposed to partition samples instead of the conventional static window partition method, and the two aspects of feature correlation and classification results were compared. For feature correlation, the average correlation coefficient between adaptive sample features and emotional state and the average correlation coefficient between static sample features and emotional state were compared. The results showed that the features extracted from the AWS had a stronger correlation with the emotional state. In terms of classification results, the AWS had better classification accuracy than static samples in a variety of machine learning methods.

In order to expand the difference between samples, we developed a fine-grained feature extraction method. Through visual analysis and verification, it was found that, after fine-grained feature extraction of samples, the extracted feature curves contained more changes and time-series information, which increased the distinction between samples and was more conducive to classification. The temporal convolutional network (TCN) and LSTM were used to mine time-series features. After fine-grained feature extraction, learners’ emotion recognition accuracy was significantly improved. We found that TCN was more suitable for emotion recognition than LSTM.

Finally, the effectiveness of the adaptive window partition method and the fine-grained feature extraction method proposed in this paper were verified again on the public dataset HCI.

In future work, the accuracy of learners’ emotion recognition could be improved by combining the semantics of videos or from the perspective of cognition. Other multi-modality fusion methods could also be used to combine eye movement features with scenario features. In addition, the waveforms of physiological signals, such as the EDA signal and PPG signal, should also be related to the fluctuation of emotion. Therefore, the adaptive window partition method and fine-grained feature extraction method could also be used to classify emotion using other physiological signals, and their effectiveness could be further verified.

## Figures and Tables

**Table 1:** The specific features of the three modalities.

Modality	Specific Features	
Eye movement signal	Time domain feature	Maximum, minimum, average, median, range, standard deviation, variance, energy, average amplitude, saccade time, fixation time and coordinate difference of eye movement, root mean square
	Wave feature	Crest factor, waveform factor, skewness factor, impulse factor, clearance factor, kurtosis factor
Audio signal	Maximum, minimum, average, median, range, standard deviation, variance	
Video image	Maximum, minimum, average, median, range, standard deviation, variance	

**Table 2:** Average correlation coefficients.

Modalities	Pupil Diameter				Audio Signal		Video Image	All the Features
	Time-domain features		Waveform features		Time-domain features		Time-domain features	
		First-order difference		First-order difference		First-order difference		
Adaptive window	0.25	0.05	0.2	0.05	0.27	0.2	0.21	0.1824
Static window	0.22	0.03	0.18	0.04	0.26	0.19	0.19	0.1656

**Table 3:** Comparison with the other adaptive window methods.

Modalities		Eye Movement			Audio			Video Image		
	Evaluation	Acc (%)	m-F1	AUC	Acc (%)	m-F1	AUC	Acc (%)	m-F1	AUC
Research										
[14]		51.3	0.46	0.7	65.2	0.6	0.76	61.8	0.56	0.77
[15]		50.2	0.46	0.69	64.9	0.59	0.75	58.1	0.53	0.75
Our method		53.7	0.48	0.71	68.4	0.63	0.82	62.4	0.58	0.77

**Table 4:** Recognition accuracy for different models with adaptive samples (n = 30) and fine-grained samples.

Sample Type	Classification Model	Modalities			
		Eye Movement (%)	Audio (%)	Video Image (%)	Feature Layer Fusion (%)
Adaptive sample	KNN	43	40	24	48
	RF	41	42	20	29
	1D-Resnet18	53.7	68.4	62.4	68.7
Adaptive fine-grained sample	LSTM	59.6	69.3	64.9	69.2
	TCN	62.9	71.1	68	71.2
	LSTM + CNN	62.3	71.9	65.7	72.2
	TCN + CNN	65.1	75.6	70.4	76.2

**Table 5:** Recognition effects of different models with 3 s SWS, AWS (*n* = 90) and fine-grained samples.

Sample Type	Classification Model	Eye Movement	Audio	Video Image
SWS	KNN	23	60	34
	RF	25	65	34
	1D-Resnet18	**27.5**	**65.3**	**35.8**
AWS	KNN	26	64	**49**
	RF	26	65	47
	1D-Resnet18	**29.5**	**66.9**	47.7
Adaptive fine-grained sample	LSTM	40.2	65.9	47.1
	TCN	41.8	66	50.2
	LSTM + CNN	40.5	66.2	45.5
	TCN + CNN	**42.** **5**	**67.1**	**52.3**

**Table 6:** Comparison with the other methods.

	Evaluation	**Acc (%)**	**m-F1**	**AUC**	**MFLOPs**	**NPs (M)**
Research						
[25]		36.2	0.31	0.49	36.78	-
[26]		33.5	0.3	0.44	0.83	20.36
[27]		40.8	0.37	0.51	2.94	159.67
Our method		42.5	0.39	0.58	3.53	171.33

## Data Availability

Not applicable.
